# Prevalence and determinants of unmet need for family planning among married women in Ghana-a multinomial logistic regression analysis of the GDHS, 2014

**DOI:** 10.1186/s40834-018-0083-8

**Published:** 2019-01-30

**Authors:** Joseph K. Wulifan, Jacob Mazalale, Christabel Kambala, William Angko, Job Asante, Stephen Kpinpuo, Albino Kalolo

**Affiliations:** 1 School of Business and Law, Department of Administration and Management Studies, University for Development Studies, Box UPW 36, Wa-campus, Wa, Ghana; 2 Department of Economics, University of Malawi, Chancellor College, P.O Box 280, Zomba, Malawi; 3 Environmental Health Department, The Malawi Polytechnic, University of Malawi, P/B 303, Chichiri, Blantyre 3, Blantyre, Malawi; 4 School of Business and Law, Department of Banking and Finance, University for Development Studies, Wa-campus, Wa, Ghana; 5 Faculty of Integrated Development Studies, Campus Officer, University for Development Studies, Wa-campus, Wa, Ghana; 6 Department of Public Health, St. Francis University College of Health and Allied Sciences, P.O.Box, 175, Ifakara, Tanzania

**Keywords:** Unmet need, Spacing, Limiting, Demographic and health survey, Multinomial regression, Ghana

## Abstract

**Background:**

Documentary evidence points to high unmet need for family planning across sub-Saharan Africa. Modern contraceptive use has been staggering over decades with unacceptable marginal increases given that one in three women still report unmet need in Ghana. This study sought to re-examine through a further analysis on the prevalence and determinants of unmet need for family planning in Ghana using married women extracted from the recent 2014 Ghana Demographic and Health Survey.

**Method:**

Data was analyzed using univariate, bivariate, logistic and multinomial logistic regression models.

**Results:**

Of the 4527 women, more than a third (35.17%) experienced unmet need of which 20.19% had unmet need for spacing while 14.98% reported unmet need for limiting. The logistic results showed that older aged women, being employed and women with higher ideal number of children were less likely to experience unmet need. However, women who did not know the couples’ preferred number of children, women who had more than one union and those with higher number of living biological children were more likely to report unmet need. From the multinomial model, an increase in age, residing in a rural area, and being employed were associated with lower risk of unmet need for spacing. Additionally, Women who did not know the couples’ ideal number of children, women who had higher age when they got married, and women with higher number of biological children were more likely to report unmet need for spacing. Women who had a higher number of ideal children, women who had secondary or higher education, women from higher socio-economic households, were less likely to report unmet need for limiting. .

**Conclusions:**

We recommend the strengthening of contraception services in order to address the various age specific needs and women within the different socio-demographic sects so as to reduce unmet need. Addressing the needs of women with increasing number of living biological children is equally paramount.

**Electronic supplementary material:**

The online version of this article (10.1186/s40834-018-0083-8) contains supplementary material, which is available to authorized users.

## Background

Globally, in 2014 alone nearly 290,000 women within the reproductive age (15–49 years) died from preventable pregnancy related complications of which sub-Saharan Africa accounted for 65% (179,000) of the deaths [1–3]. Women from low and middle income countries-LMICs are consistently more likely (1 in 30) to die of pregnancy and unsafe abortion related complications compared to (1 in 9500) women from the developed world [3]. Adherence to contraceptive use has witnessed a significant reduction in maternal mortality rate by 40% in LMICs over the last two decades [4]. Contraception adoption invariably lengthens inter-pregnancy intervals and ultimately improves child survival and maternal health outcomes [3]. A conscious management of one’s desired fertility can improve household scarce resources and family welfare [5].

Most family planning interventions aim at reducing unmet need for modern contraception which has been the fundamental cause of unintended pregnancies. Following the Ghana demographic and health survey (GDHS, 1993), nearly 40% of married women reported unmet need for family planning and by 2014, the GDHS reported an unmet need of 30%. This means the change over the two decades has been very slow than previously anticipated [6]. Conceptually, scholars define unmet need for family planning to encompass the discrepancy between a woman’s reproductive intensions and contraception behaviour [7–10]. Unmet need arises from two categories of women-thus pregnant women whose pregnancies were mistimed or unwanted because they were not using any contraception method before the pregnancy and fertile non-pregnant women who currently are not using any method of contraception who do not intend to give birth in the next 2 years or are undecided whether they wanted another pregancy [7, 11]. A woman is said to have unmet need for family planning if desired to delay the next child birth by at least 2 years but no using any method of family planning [12]. A pregnant woman has unmet need if she desired to have had her current pregnancy later (unmet need for spacing/mistimed pregnancy) or did not want the pregnancy at the time she got pregnant (Unmet need for limiting/unwanted pregnancy) but retrospectively did not use any contraception before the pregnancy [13]. The non-pregnant fertile woman has unmet need if she equally prefers to have the next child in at least 2 year’s time (unmet need for spacing), isn’t sure when to have the next child (unmet need for spacing) or reports not wanting any more children (unmet need for limiting) but using any contraception [7]. Given the high failure rate associated with traditional methods, women who use traditional method of contraception are regarded as having unmet need for family planning [14].

In Ghana, contraceptive prevalence rate (CPR) among currently married women of reproductive age rose from 10% each for traditional and modern methods of contraception to 7 and 22% respectively in 2014 [15]. Unmet need for family planning has important public health concerns as it often associated with unintended pregnancies [16]. Unintended pregnancies carry serious consequences for the woman and their families including the likelihood of unsafe abortion, delayed prenatal care, poor maternal and child health outcomes [16, 17]. Till date, it is still not very clear which single factor(s) operate to influence the persistent low contraceptive use and the corresponding high unmet need given that one in three women still report unmet need for contraception in Ghana [6, 18]. In recent years, unmet need for family planning has gained prominence in policy and academia hence its inclusion in the just ended millennium development goals (MDGs 2015) and by extension the space it occupies in the proposed sustainable development goals (SDGs 2030) [19, 20]. Currently there is no study that examined unmet need for modern contraception among currently married women in Ghana at the three analytical levels using the recent 2014 DHS data. Given this background, an assessment of the individual, household and facility factors that are associated with unmet need for family planning is not only timely but also appropriate. Our study therefore sought to examine which factors are associated with unmet need among married women in Ghana looking specifically at the levels of unmet need for spacing, limiting as well as the characteristics of women with unmet need for contraception.

## Methods

### Data extraction

We used the 2014 Ghana Demographic and Health Survey (GDHS) data for this manuscript. The data was extracted from the Measure DHS web platform after explicit permission sought to use the data was granted by the data originators (https://dhsprogram.com/). The entire survey covered a sample of 9396 women of reproductive age (15–49 years). The survey employed multi-stage sampling technique. Details of the data sampling techniques, data collection procedures, research assistant’s training and pre-test details are outlined in the report [15]. The primary purpose of the GDHS was to generate recent and reliable data on fertility, family planning, maternal and child health, infant and child mortality and nutrition [21].

### Study design

The 2014 GDHS was cross-sectional in design in which men and women who met the survey criteria were interviewed using a structured questionnaire. The analyses in this manuscript was restricted to currently married women of reproductive ages (15–49 years), fecund but reported their desire to space childbirth for at least 2 years or limit childbirth to their desired number but were not using any contraceptive method. In our analysis, we excluded women who had never had sexual intercourse, infecund and menopausal women. Given this criteria, we reduced the number of women to 4527 of which 1592 reported unmet need for family planning.

### Study variables

The outcome variables for this study, which was unmet need for family planning, was derived from a series of specific questions in the section under contraception in the individual woman’s questionnaire. Unmet need was defined as a dichotomous variable, differentiating between women with unmet need for family planning (coded as 1) and women without unmet need (coded as 0). Women in the non-contraceptive users’ category were separated into pregnant women and non-pregnant women. A pregnant woman was defined as having unmet need if she indicated that her pregnancy was either wanted later (mistimed pregnancy) or she did not want to be pregnant (unwanted pregnancy) but was not using any method of contraception before the pregnancy. Women with mistimed pregnancies were classified as having unmet need for spacing while those with unwanted pregnancies were classified as having unmet need for limiting. At the non-pregnant fertile women category, women were further separated into women who desired to have a child in at least after 2 years (unmet need for spacing), women who were not sure when to have their next child (unmet need for spacing) and women who indicated they didn’t want additional children (unmet need for limiting). The two categories of women from pregnant and fertile non-pregnant women together (unmet need for spacing and unmet need for limiting) are referred to total women with unmet need for family planning consistent with prior scholarly studies [7, 8, 10–12].

The independent variables were selected for inclusion in the analysis based on their significance in prior studies on factors which influence unmet need for family planning. These variables were identified under four categories comprising demographic, socio-eco-cultural, proximate, programme and health facility related. All variables included in the analysis were carefully selected from the women’s questionnaire. For meaningful analysis and interpretation, a few of the variables were regrouped from their original categories in the main dataset which are explained in the subsequent paragraphs consistent with prior studies [22].

The demographic variables included were age of woman, age at first marriage and number of living children. Age was regrouped into the conventional five standard age groupings as 15–19, 20–24, 24–29, 30–34, 35–39, 40–44 and 45–49. Age at first marriage and number of living children maintained their original continuous variables structure. The socio-eco-cultural variables included in the analysis were religion, number of unions, residence, woman’s level of education, household wealth, occupation and status of been registered under the national health insurance scheme. Religion was regrouped into Christianity, Islam and others/no religion [23]. Variables related to programme and health facility included sources from which individual heard information on family planning (radio, T.V and newspapers), whether the individual visited a health facility in the prior 12 months, whether the individual was visited by a health worker and if the individual knows a modern family planning method.The last category of variables which most prior studies classify as proximate/attitudinal variables [11, 22] included couple desire for children and ideal number of children. Table one presents the variables, their measurements and our hypothesis on association with unmet need.

### Analytical approach

We analyzed the data at three (3) levels. At the first level we employed univariate analysis which included an assessment of the level of unmet need, and a sample description using the demographic, socioeconomic proximate and programme/facility variables. Frequencies and percentages were used to guide the sample description. The second level applied bivariate analysis which focused on establishing the unadjusted relationship between the explanatory variables and the outcome variable - unmet need. This approach provided a preliminary assessment of unmet need levels across the individual explanatory variables and a further overview of the characteristics of women with unmet need for contraception. The third level of analysis focused on multivariate analysis to assess adjusted association of the explanatory variables with unmet need. We used a logistic regression model and the multinomial logistic regression model at the multivariate level to assess how the factors are associated with unmet need. The binary logistic regression was used because the outcome variables for total unmet need was dichotomous (no unmet need coded as 0, and unmet need coded as 1). The multinomial logistic regression was applied because the second categorical dependent variable of unmet need had three categories (no unmet need coded as 1, unmet need for spacing coded as 2, and unmet need for limiting coded as 3).

The multivariate analysis was based on an accurate representative of the population from which the sample was taken. The dichotomous relationship between unmet need for family planning and the various explanatory variables were analyzed using the logistic regression. The multinomial regression model was used to examine the association between selected explanatory variables on unmet need for spacing and unmet need for limiting. This was possible because the outcome variables had three categories (spacing, limiting and met need). This means that two dichotomies were compared (unmet need for limiting to no unmet need and unmet need for spacing to no unmet need) rather than one.

## Results

### Univariate analysis

Table 1 reports the distribution of selected background characteristics of respondents. A total of 4527 women were interviewed with 35.17% women reporting unmet need which was further split into unmet need for spacing (20.19%) and unmet need for limiting (14.98%). Majority of the women (23.0%) were found between 25 and 29 years while the least proportions of women were in age categories 15–19 and 45–49 years (3.0 and 6.0%) respectively. Pentecostal Protestants formed the majority (36.0%) of the religious sect and the minority (7.0%) being respondents without a religion or practised traditional religion. Majority of women (79.0%) belonged to the category of women who reported ever been engaged in a single union compared to their counterparts (21.0%) who had more than one marriage unions. Majority of the respondents were from rural areas. On educational status, majority (47.0%) had at least secondary education. Women from the poorest households formed a third (30.0%) while women from the richest category were (16.0%). The proportion of women with employment status was the highest (83.0%) as against 17.0% who reported not having an employment. Pertaining to information on family planning, 57.0% women reported they heard of family planning from the radio, 56.0% mentioned T.V while 5.0% identified the newspaper. 64.0% of the women reported having visited a health facility in the past 12 months while 14.0% confirmed also been visited by a health worker. The number of women who knew a family planning method was almost universal (99.0%). Majority of women (70.0%) reported being subscribed members under the Ghana national health insurance scheme (NHIS). The bivariate are presented in Table 2 in the appendix.

**Table 1:** Variable measurements and their respective expected sign on unmet need

Variable	Measurement	*n* = 4527	%	Hypothesized/expected coefficient
Age				
	1 = 15–19	118	3	+
	2 = 20–24	632	14	
	3 = 25–29	1038	23	
	4 = 30–34	967	21	
	5 = 35–39	891	20	
	6 = 40–44	592	13	
	7 = 45–49	289	6	
Religion				
	0 = no or traditional. religion	314	7	+
	1 = catholic	651	14	
	2 = non-Pentecostal protestant	987	22	
	3 = Pentecostal protestant	1614	36	
	4 = Islam	961	21	
Number of unions ever engaged in				
	1 = once	3557	79	–
	2 = more than once	970	21	
Residence				
	1 = urban	1995	44	–
	2 = rural	2532	56	
Education				
	0 = no education	1528	34	+
	1 = primary	868	19	
	2 = secondary +	2131	47	
SES				
	1 = poorest	1339	30	+
	2 = poorer	878	19	
	3 = middle	823	18	
	4 = richer	768	17	
	5 = richest	719	16	
Occupation				
	0 = no	779	17	+
	1 = yes	3748	83	
Heard FP methods on the radio				
	0 = no	1951	43	+
	1 = yes	2576	57	
Watched FP messages on the TV				
	0 = no	2550	56	+
	1 = yes	1977	44	
Read FP messages in newspapers				
	0 = no	4314	95	+
	1 = yes	213	5	
Woman visited a health facility in the past 12 months				
	0 = no	1617	36	+
	1 = yes	2910	64	
FP worker visited woman				
	0 = no	3879	86	+
	1 = yes	648	14	
Woman knows modern FP methods				
	0 = doesn’t know modern FP methods	50	1	+
	1 = knows modern FP methods	4477	99	
Couple’s congruence of desire for children				
	1 = both want same	2101	46	–
	2 = husband wants more	1178	26	
	3 = husband wants fewer	402	9	
	8 = don’t know	846	19	
Woman is member of any health insurance				
	0 = no	1350	30	+
	1 = yes	3177	70

**Table 2:** Descriptive statistics

	Univariate			Bivariate											
	Overall sample (*n* = 4527)			Women with unmet need - total (*n* = 1592) = 35.17%				Women with unmet need for spacing (*n* = 914) = 20.19%				Women with unmet need for limiting (*n* = 678) = 14.98%			
Variable	Measurement	n	%	n	%	Chi2	*p*-value	n	%	Chi2	*p*-value	n	%	Chi2	*p*-value
Age															
	15–19	118	3	56	47	77	0.00	54	47	96	0.00	2	3	451	0.00
	20–24	632	14	201	32			189	30			12	3		
	25–29	1038	23	306	29			265	27			41	5		
	30–34	967	21	295	31			180	21			115	15		
	35–39	891	20	337	38			153	22			184	25		
	40–44	592	13	260	44			59	15			201	38		
	45–49	289	6	137	47			14	8			123	45		
Religion															
	None or traditional religion	314	7	118	38	7	0.14	66	25	5	0.25	52	21	31	0.00
	Catholic	651	14	212	33			131	23			81	16		
	Non-Pentecostal protestant	987	22	352	36			169	21			183	22		
	Pentecostal protestant	1614	36	594	37			328	24			266	21		
	Islam	961	21	316	33			220	25			96	13		
Number of unions															
	One	3557	79	1191	33	21	0.00	743	24	0	0.65	448	16	68	0.00
	More than one	970	21	401	41			171	23			230	29		
Residence															
	Urban	1995	44	686	34	1	0.33	398	23	0	0.58	288	18	1	0.32
	Rural	2532	56	906	36			516	24			390	19		
Education															
	no education	1528	34	544	36	4	0.15	318	24	3	0.19	226	19	2	0.45
	primary	868	19	326	38			187	26			139	20		
	secondary +	2131	47	722	34			409	22			313	18		
SES															
	Poorest	1339	30	450	34	23	0.00	284	24	12	0.02	166	16	23	0.00
	2	878	19	355	40			195	27			160	23		
	3	823	18	314	38			170	25			144	22		
	4	768	17	250	33			139	21			111	18		
	Least poor	719	16	223	31			126	20			97	16		
Occupation															
	Unemployed	779	17	301	39	5	0.03	225	32	32	0.00	76	14	11	0.00
	Employed	3748	83	1291	34			689	22			602	20		
Heard FP messages on radio															
	No	1951	43	706	36	2	0.21	418	25	3	0.08	288	19	0	0.98
	Yes	2576	57	886	34			496	23			390	19		
Heard FP messages on TV															
	No	2550	56	933	37	5	0.02	538	25	4	0.05	395	20	2	0.13
	Yes	1977	44	659	33			376	22			283	18		
Read FP messages in newspaper															
	No	4314	95	1529	35	3	0.08	880	24	3	0.09	649	19	1	0.37
	Yes	213	5	63	30			34	18			29	16		
You visited health facility in the past 12 months															
	No	1617	36	605	37	6	0.02	326	24	0	0.51	279	22	11	0.00
	Yes	2910	64	987	34			588	23			399	17		
FP worker visited you in the past 12 months															
	No	3879	86	1354	35	1	0.37	766	23	3	0.10	588	19	0	0.64
	Yes	648	14	238	37			148	27			90	18		
Knows modern FP methods															
	No	50	1	14	28	1	0.29	10	22	0	0.75	4	10	2	0.15
	Yes	4477	99	1578	35			904	24			674	19		
Couple’s desire for children															
	Both want the same	2101	46	678	32	20	0.00	385	21	21	0.00	293	17	6	0.09
	Husband wants more children	1178	26	419	36			231	23			188	20		
	Husband wants fewer children	402	9	150	37			84	25			66	21		
	Does not know	846	19	345	41			214	30			131	21		
Membership to any health insurance scheme															
	Not member	1350	30	505	37	4	0.04	266	24	0	0.86	239	22	11	0.00
	Member	3177	70	1087	34			648	24			439	17		
Continuous variables															
Overall sample (*n* = 4527)				Total unmet need (*n* = 1592)				Unmet need for spacing (*n* = 1154)				Unmet need for limiting (*n* = 696)			
		mean	Sd	mean	sd	t-statistic	p-value	mean	sd	t-statistic	*p*-value	mean	sd	t-statistic	*p*-value
Age at first marriage	Yes	19.72	5	19.34	4.34	4.07	0.00	19.46	4	3	0.01	19.17	4	4	0.00
	No			19.2	4.7			19.92	5			19.92	5		
Number of living biological children	Yes	3.13	2	3.63	2.04	−12.78	0.00	2.66	2	3	0.00	4.94	2	−26.08	0.00
	No			2.86	1.90			2.86	2			2.86	2		
Ideal number of children	Yes	4.44	1	4.43	1.25	0.62	0.54	4.41	1	1	0.37	4.45	1.31	−0.01	0.99
	No			4.45	1.26			4.45	1			4.45	1.26		

### Bivariate analysis

The bivariate distribution of currently married women who reported unmet need for contraception according to selected background characteristics are presented in Table 2. The Pearson’s Chi-square test reported statistically significant variation in age of woman and total unmet need (X^2^ = 77; *P* < 0.001), unmet need for spacing (X^2^ = 96; P < 0.001) and unmet need for limiting (X^2^ = 451; P < 0.001). There was a significant difference between woman’s religion and unmet need for limiting (X^2^ = 31; *P* < 0.001) but no significant variation was observed between woman’s religion and total unmet need as well as unmet need for spacing. Significant difference was observed in number of unions and total unmet need (X^2^ = 21; *P* < 0.001) as well as unmet need for limiting (X^2^ = 68; *P* < 0.001) but not unmet need for spacing. On socioeconomic status, the results showed a significant difference between household wealth and all the three levels of unmet need (total unmet need, unmet need for spacing and unmet need for limiting). The results equally showed a significant difference in woman’s occupation and the three levels of unmet need. Statistically significant variation was observed between women who heard of family planning message on the T.V and total unmet need (X^2^ = 5; *P* < 0.002) as well as unmet need for spacing (X^2^ = 4; *P* < 0.005). Statistically significant variation was equally observed between women who visited a health facility in the past 12 months and both total unmet need (X^2^ = 6; *P* < 0.002) as well as unmet need for limiting (X^2^ = 11; *P* < 0.001). We observed a statistical significant change in the proportion of women with both total unmet need (X^2^ = 20; *P* < 0.001) and unmet need for spacing (X^2^ = 21; *P* < 0.001) by couple’s desire for children but not unmet need for limiting. Significant variation was observed between women who subscribed to the Ghana National health insurance scheme (NHIS) and both total unmet need and unmet need for limiting. The t-test results detected a significant difference between mean age of woman at first marriage and total unmet need (*t* = 4; *P* < 0.001), unmet need for spacing (*t* = 3; *P* < 0.001) and unmet need for limiting (t = 4; *P* < 0.001). Lastly, we also observed a significant difference between number of living biological children and the three levels of unmet need for family planning.

### Multinomial regression results

Table 3 presents the logistic regression (Model I) estimates of odds ratio and the multinomial regression (Model II) estimates of relative risk ratios (RRR) for selected variables of currently married women on the three levels of unmet need (total unmet need, unmet need for spacing and unmet need for limiting). Model I reveals that for total unmet need, women with higher age were less likely to report unmet need for family planning compared to women aged 15–19 years. No significant difference was detected across the various religious faiths. Women who had more than one marriage union were more likely (OR = 1.21; CI 1.03–1.42) to report unmet need for family planning compared with their colleagues who had one marriage union. No significant difference was observed across socioeconomic status. Only women from second quintile were more likely to report unmet need (OR = 1.28; CI 1.05–1.56) compared with women from poorest households. Women who reported having employment were less likely (OR = 0.79; CI 0.66–0.93) to report unmet need compared to their colleagues who had no employment. Couple who did not know the preferred number of children were more likely (OR = 1.37; CI 1.14–1.66) to experience unmet need compared to women who reported the couple desired the same number. Women who had an additional living biological child were more likely (OR = 1.36; CI 1.28–1.43) to report unmet need. Also, women who had an additional ideal child were less likely (OR = 1.86; CI 1.81–1.92) to experience unmet need.

**Table 3:** Determinants of unmet need for family planning

Regression results (*n* = 4527)										
Variable and measurement		Model I [Logistic]			Model II [Multinomial]					
		Women with unmet need-total (*n* = 1592)			Women with unmet need for spacing (n = 914)			Women with unmet need for limiting (n = 678)		
		OR	95% CI		RRR	95% CI		RRR	95% CI	
Age										
	15–19	1			1			1		
	20–24	0.448***	0.296	0.680	0.481***	0.314	0.736	0.634	0.140	2.875
	25–29	0.332***	0.218	0.506	0.376***	0.242	0.586	0.882	0.207	3.757
	30–34	0.255***	0.163	0.399	0.251***	0.155	0.406	1.557	0.373	6.491
	35–39	0.284***	0.177	0.456	0.231***	0.137	0.390	2.23	0.527	9.430
	40–44	0.279***	0.171	0.456	0.132***	0.075	0.233	2.88	0.683	12.150
	45–49	0.308***	0.180	0.530	0.065***	0.031	0.140	3.98	0.930	17.030
Religion										
	none or trad. Religion	1			1			1		
	Catholic	0.924	0.692	1.234	0.891	0.652	1.219	0.89	0.545	1.453
	Non-Pentecostal protestant	0.952	0.716	1.266	0.787	0.569	1.088	1.159	0.726	1.849
	Pentecostal protestant	1.076	0.829	1.396	0.974	0.732	1.294	1.172	0.748	1.838
	Islam	0.968	0.729	1.283	0.967	0.720	1.299	0.768	0.457	1.290
Number of unions										
	One	1			1			1		
	More than one	1.212*	1.033	1.423	1.214	0.992	1.486	1.204	0.960	1.510
Residence										
	Urban	1			1			1		
	Rural	0.831	0.687	1.007	0.784*	0.630	0.977	0.892	0.661	1.203
Education										
	no education	1			1			1		
	primary	1.034	0.856	1.249	0.942	0.758	1.172	1.143	0.845	1.546
	secondary +	1.099	0.910	1.327	0.888	0.710	1.112	1.531**	1.138	2.060
SES										
	Poorest	1			1			1		
	2	1.284*	1.057	1.561	1.191	0.955	1.485	1.627**	1.195	2.216
	3	1.242	0.974	1.583	0.991	0.756	1.300	2.012***	1.367	2.961
	4	1.042	0.786	1.383	0.804	0.574	1.126	1.856**	1.186	2.904
	Least poor	1.077	0.777	1.493	0.848	0.572	1.258	1.809*	1.096	2.986
Occupation										
	Unemployed	1			1			1		
	Employed	0.789**	0.666	0.934	0.707***	0.592	0.845	0.964	0.703	1.320
Heard FP messages on radio										
	No	1			1			1		
	Yes	0.933	0.797	1.093	0.949	0.793	1.136	0.918	0.717	1.176
Heard FP messages on TV										
	No	1			1			1		
	Yes	0.974	0.814	1.164	1.00	0.812	1.232	0.912	0.702	1.185
Read FP messages in newspaper										
	No	1			1			1		
	Yes	0.974	0.722	1.313	0.788	0.528	1.177	1.393	0.920	2.107
You visited health facility in the past 12 months										
	No	1			1			1		
	Yes	0.883	0.767	1.017	0.872	0.737	1.032	0.93	0.752	1.150
FP worker visited you in the past 12 months										
	No	1			1			1		
	Yes	1.101	0.915	1.326	1.205	0.971	1.494	1.017	0.769	1.343
Knows modern FP methods										
	No	1			1			1		
	Yes	1.158	0.556	2.415	1.428	0.664	3.070	0.861	0.259	2.865
Couple’s desire for children										
	Both want the same	1			1			1		
	Husband wants more children	1.081	0.923	1.267	1.093	0.905	1.320	1.094	0.850	1.408
	Husband wants fewer children	1.214	0.948	1.555	1.289	0.975	1.704	1.176	0.813	1.702
	Does not know	1.376***	1.141	1.660	1.5***	1.225	1.837	1.134	0.823	1.561
Membership to any health insurance scheme										
	Not member	1			1			1		
	Member	1.006	0.871	1.161	1.002	0.845	1.188	1.005	0.802	1.258
Age at first marriage		1.008	0.990	1.025	1.023*	1.001	1.045	0.98	0.956	1.004
Number of living biological children		1.36***	1.286	1.438	1.111**	1.038	1.189	1.775***	1.636	1.927
Ideal number of children		0.863***	0.810	0.920	0.957	0.888	1.032	0.722***	0.656	0.795

*** p < 0.001, ** p < 0.01, * p < 0.05

In Model II, women with higher age were relatively less likely to report unmet need for spacing compared to women within age group 15–19 years. No significant difference was observed across the age categories and unmet need for limiting. Women from rural areas were relatively less likely (RRR = 0.78; CI 0.63–0.97) to report unmet need for spacing compared to their colleagues from urban areas. Women who had at least secondary education were relatively more likely (RRR = 1.53; CI 1.13–2.06) to report unmet need for limiting compared to women who had no education. Women from higher socioeconomic status households were relatively more likely to report unmet need for limiting compared with women from poorest households. Women who were employed were relatively less likely (RRR = 0.70; CI 0.59–0.84) to report unmet need for spacing compared to unemployed women. Couple who reported they did not know the desired number of children were more likely (RRR = 1.50; CI 1.22–1.83) to report unmet need for spacing relative to couple who both want the same number of children. From the continuous variables, women who had an additional age at first marriage were relatively more likely (RRR = 1.02; CI 1.00–1.04) to report unmet need for spacing. Women who had an additional number of living biological child were relatively more likely to report both unmet need for spacing (RRR = 1.11; CI 1.03–1.19) and unmet need for limiting (RRR = 1.77; CI 1.63–1.92). Finally women who had an additional ideal child were relatively less likely (RRR = 0.72; CI 0.66–0.80) to report unmet need for limiting.

## Discussion

This study assessed the associations between selected explanatory variables and unmet need in Ghana. The patterns of high unmet need as reported in unmet need prevalent country like ours (35.17%) reveals that, unmet need increased among Pentecostal Protestants, women who had more than one marriage unions, women who did not know couple’s desired number of children and women who had an additional number of living biological child. Unmet need decreased among women as they grew older, among those employed and women with an additional number of ideal child. Unmet need for spacing was positive among women who did not know couple’s desired number of children, as the age of women getting married increases and women who had additional living biological child. Unmet need for spacing decreased as women grew older, among rural women and women employed. Unmet need for limiting was positive among women who had at least secondary education, women from wealthier households and among women with an additional living biological child. Unmet need for limiting decreased as women had an additional ideal child.

The results suggest that unmet need for spacing and total unmet need were negatively associated with age. As women grew older, both unmet need for spacing and total unmet need decreased but no significant association was observed across ages under unmet need for limiting. This is expected in that, younger mothers might have not reached the desired fertility and hence preferred to space childbirths than limit [11, 24]. These findings are consistent with three other studies from SSA focusing on women’s fertility choices. These studies posit that, whereas the need for spacing childbirth was more likely not to be met among younger women who had not yet achieved their desired fertility goals and for that matter would be more interested in spacing future pregnancies, the unmet need for limiting was negative during later reproductive years once their desired family size was achieved and no further pregnancies are desired [25–27]. It will therefore be beneficial for policies targeting unmet need to address women according to their age specific needs.

We anticipated that given the underlying Catholic doctrines, Catholics compared to traditional religion were expected to be more likely to report unmet need but the results pointed no significant difference across the various religious faiths. In the current study, Pentecostal Protestants women had higher odds of unmet need compared to traditional religion but was equally not statistically significant. In a prior study, Muslim women had lower odds of unmet need compared to orthodox Christians and this might be attributable to the tendency of Muslim women to have higher total fertility compared to Christians [28]. In a reviewed study on determinants of unmet need from selected studies in LMICs the influence of religious beliefs on family planning decisions appeared a little complex [11]. Data from six quantitative studies examining the role of a woman’s religious belief detected significant associations with unmet need for contraception [11]. In a Nepal study, Muslim belief was positively associated with an increase in unmet need when compared to Hinduism [29]. In another study on unmet need in Ghana, Catholicism was identified to be negatively associated with unmet need when compared to other religious sects [30]. A qualitative study from Uganda on determinants of unmet need findings were consistent as they identified Anglicans to be more positive towards contraceptive use compared to Muslims and Catholics [11, 31]. In an Indian study for instance, Hindu traditions identified selected menstrual taboos amongst which required relatives close observation of a woman’s fertility and thus made contraceptive use limited [32].

Our study detected that unmet need was dominant among women who had more than one marriage unions. A study reported that, marital instability can potentially propel women to re-marry and have additional children from the new matrimonial home hence increasing the woman’s total fertility beyond her initial fertility intensions given that the initial marriage was note terminated [33]. The desire to have additional children in the new matrimonial home can trigger unmet need for contraception. Consistent with this finding, a review of selected studies on Male attitude and behavior on family planning in Africa reported that new unions propel higher parity progression since in the African context, it is believed that any marriage should be graced with at least a child to symbolize effective marriage union even if the woman had given birth to her desired number of children in the previous marriage [34, 35].

Place of residence did not exhibit any significance for total unmet need and unmet need for limiting except for only unmet need for spacing where rural dwellers were less likely to report unmet need in the multinomial analysis. The outcome is inconsistent with prior studies in Uganda and Ethiopia which reported higher odds on unmet need among rural women [22, 36]. The lower levels of unmet need relative to their urban counterparts is again inconsistent with another Ethiopian study in the fact that rural women relatively have lower decision making power on contraceptive use and could not report substantial contraceptive use level to reflect the reduction in unmet need reported [28].

Our multinomial results detected that women with at least secondary education were more likely to report unmet need for limiting. Unmet need is often higher among women with a primary school education. This is because women with higher education are relatively more likely to report contraceptive use and women with no education generally want more children [33, 37]. This results are inconsistent with results from Burkina Faso and Malawi which reported higher likelihood of unmet need among rural women with lower educational levels [16, 33].

Our model I did not detect any significant levels across the socioeconomic status except for our Model II which reported positive association with unmet need for limiting as household wealth increases. Higher unmet need for limiting was reported among the less poor households. Unlike in most prior studies where lower wealth quintiles associated positively with unmet need for contraception [22, 28], our findings reported a contradiction in that women from higher wealth quintiles were more likely to report unmet need against the view that women with wealthier background ought to have better decision making power on contraceptive use and should therefore report lower levels of unmet need [11, 28, 33].

Woman’s occupational status was identified to be negatively associated with unmet need and unmet need for spacing. Previous studies suggests that female occupation and an independent income positively enhances a woman’s autonomy by improving financial decision-making within a household which can lead to better contraceptive use decision [38, 39]. Also professionals/ clerical occupations workers were observed in a Zambian study to be significantly less likely to report unmet need for spacing compared to their non-working colleagues, where as all other occupations except those in Agriculture were equally significantly less likely to report unmet need for limiting compared to their unemployed companions [24]. This means that there were differences in contraceptive use among the different occupational groupings. Given that woman’s autonomy in employment can contribute to an increase in the contraceptive use and thus a reduction in unmet need, policies should focus on assisting women acquire gainful employment.

Total unmet need and unmet need for limiting were negative among married women who desired an additional child. This trend was expected as some of the currently married women who mentioned and additional child as their ideal number might have not had this desired number either presently or in the past and are less willing to limit child bearing but have no access to consistent modern contraceptive use. Alternatively previous studies attest that, their colleagues who desired additional children were probably in active child bearing and for that matter might prefer contraception to space future childbirths [33]. In rural settings in Ghana like many parts in SSA, contraception to limit childbearing is scarce compared to other modern methods which are more available. Long acting contraceptive method use decisions are difficult to reach than other methods [14]. Our findings are consistent with previous studies which equally suggest lower unmet need detected among married women who reported higher number of children as their ideal preference [40].

### Methodological considerations

The study population consisted of currently married fertile women at the time of the study. This means that the prevalence of unmet need should not be misconstrued and generalized to cover the entire population of women in Ghana as the method for calculation did not cover all women. Following the objective of our study, we carefully selected the category of currently married women who are consistently referred to in literature as being at extremely higher risk of unmet need [7]. Also given the cross-sectional design nature of the study, all variables included in the regression analysis were information obtained at the time of the survey which only provides evidence of statistical associations between the predictor and dependent variables and cannot show cause-effect relationship.

## Conclusion

The results showed that an increase in age, residing in a rural area, and being employed were associated with lower risk of unmet need for spacing. Additionally, Women who did not know the couples’ ideal number of children, women who had higher age when they got married, and women with higher number of biological children were more likely to report unmet need for spacing. Women who had a higher number of ideal children, women who had secondary or higher education, women from higher socio-economic households, were less likely to report unmet need for limiting.

The unmet need for contraception reported in this study is high compared to other LMICs with similar socio-demographic characteristics given the more than one in three married women reporting unmet need is a major concern. The study recommend that continuous health education on the significance of family planning both at the individual and household level is an important strategic measure to adopt if contraceptive use decisions are to be enhanced so as to reduce the lagging persistent unmet need for family planning in Ghana. Community based family planning programmes should be strengthened and expanded to serve all rural communities. A recent study in Ethiopia reported majority of contraceptive users preferred child spacing to limiting which supports many studies in SSA and by extension Ghana [41, 42]. The findings are crucial and points the need for programmes to incorporate access to contraceptive services and raise educational levels to bring about behavioral and attitudinal change among the affected women so as to lower the nagging desire for additional children. Specific emphasis should be accorded to educated women and women within the various socioeconomic levels. Qualitative research is needed to understand the reasons for the underlying trend.

## Additional files

Additional file 1: Tabulation of unmet_need_tu. (XLS 1710 kb)

Additional file 2: Results log. (LOG 3 kb)

## Abbreviations

CI: Confidence interval
FP: Family planning
GDHS: Ghana demographic health survey
LMICs: Low and middle income countries
NHIS: Nation health Insurance scheme
OR: Odds ratio
RRR: Relative risk ratio
SES: Socioeconomic status
SSA: Sub-Saharan Africa
T.V: Television
